# Induction of Antihuman C–C Chemokine Receptor Type 5 Antibodies by a Bovine Herpesvirus Type-4 Based Vector

**DOI:** 10.3389/fimmu.2017.01402

**Published:** 2017-10-25

**Authors:** Andrea Elizabeth Verna, Valentina Franceschi, Giulia Tebaldi, Francesca Macchi, Valentina Menozzi, Claudia Pastori, Lucia Lopalco, Simone Ottonello, Sandro Cavirani, Gaetano Donofrio

**Affiliations:** ^1^Department of Medical Veterinary Science, University of Parma, Parma, Italy; ^2^Division of Immunology, Transplantation and Infectious Diseases, San Raffaele Scientific Institute, Milan, Italy; ^3^Biochemistry and Molecular Biology Unit, Laboratory of Functional Genomics and Protein Engineering, Department of Life Sciences, University of Parma, Parma, Italy

**Keywords:** bovine herpesvirus 4, gene delivery vector, human CCR5, recombinant virus, antihuman CCR5 antibodies

## Abstract

Bovine herpesvirus 4 (BoHV-4) is a promising vector for the delivery and intracellular expression of recombinant antigens and can thus be considered as a new prototype vaccine formulation system. An interesting, and actively pursued, antigen in the context of human immunodeficiency virus (HIV) infection prophylaxis (and therapy) is the C–C chemokine receptor type 5 (CCR5) co-receptor, whose blockage by specific antibodies has been shown to inhibit both viral entry and cell-to-cell transmission of the virus. Building on our previous work on the BoHV-4 vector system, we have engineered and tested a replication-competent derivative of BoHV-4 (BoHV-4-CMV-hCCR5ΔTK) bearing a human CCR5 (hCCR5) expression cassette. We show here that CCR5 is indeed expressed at high levels in multiple types of BoHV-4-CMV-hCCR5ΔTK-infected cells. More importantly, two intravenous inoculations of CCR5-expressing BoHV-4 virions into rabbits led to the production of anti-CCR5 antibodies capable of reacting with the CCR5 receptor exposed on the surface of HEK293T cells through specific recognition of the amino-terminal region (aa 14–34) of the protein. Given the growing interest for anti-CCR5 immunization as an HIV control strategy and the many advantages of virus-based immunogen formulations (especially for poorly immunogenic or self-antigens), the results reported in this study provide preliminary validation of BoHV-4 as a safe viral vector suitable for CCR5 vaccination.

## Introduction

Bovine herpesvirus 4 (BoHV-4) is dsDNA genome virus belonging to *Herpesviridae* family, *Gammaherpesvirus* subfamily and *Rhadinovirus* genus. The natural host for BoHV-4 is the bovine; however, BoHV-4 presence has been detected from many other animal species. One of BoHV-4 features is its ability to replicate *in vitro* both in primary cultures and cell lines from various animal species (1–7), and, *in vivo*, to experimentally infect many non-natural hosts such as mice (5, 8, 9), rats (10), rabbits (4), sheep (2), swine (11), and goats (7).

*Ex vivo* infection of non-human primate tissue explants has also been observed (paper in preparation). This feature suggests the use of BoHV-4 as a potentially competent viral vector for *in vivo* human cell transduction as well. In contrast to other gamma-herpesviruses, there are neither growth-transformation signals nor any virus-related pathology within the natural or experimental hosts of BoHV-4. Recombinant BoHV-4, derived from the cloned BoHV-4 genome in the form of a bacterial artificial chromosome (BAC), able to express immune-dominant antigens derived from different pathogens was successfully used for immunization purposes in the abovementioned non-natural host species without any apparent detrimental effect, overt clinical sign or pathology causally related to viral vector inoculation (2, 4, 5, 7–11). Furthermore, evident oncolytic properties in immune-competent orthotopic syngeneic mouse and rat glioma models were correlated to the herpes simplex virus-1 thymidine kinase (HSV-1-TK) gene included into BoHV-4-based vector genome (6).

The C–C chemokine receptor type 5 (CCR5) is not only involved in leukocyte trafficking during inflammatory processes but also serves as a major entry site and co-receptor for human immunodeficiency virus (HIV) internalization and thus contributes to intercellular spreading of the virus (12). The multiple and overlapping (partially redundant) interactions between chemokines and their receptors make CCR5 largely dispensable in various contexts. This is reflected by the generally good health of subjects homozygous for a 32 bp deletion in the coding sequence of CCR5 (*CCR5*Δ32) that renders the protein dysfunctional (12). Because of its role as a virus co-receptor, CCR5 represents a very attractive target for preventing/controlling HIV infection and multiple anti-HIV strategies centered on this receptor are being developed (13). These include small-molecule CCR5 antagonists approved for clinical use such as Aplaviroc (GlaxoSmithKline), Maraviroc (Pfizer), and Vicriviroc (Schering-Plough). A second approach, inspired by the protective effects documented for natural as well as virus exposure-induced anti-CCR5 antibodies (13–16), relies on anti-CCR5 vaccination as an innovative anti-HIV strategy capable of providing effective protection or, at least, reduced viral replication and spreading of the infection. Importantly, naturally occurring or immunization-induced anti-CCR5 antibodies have been shown to be capable of multi-clade human papillomavirus blockage, a result that is rarely achieved with the use of conventional HIV-based immunogens (17–21).

Despite the promising aspects of therapeutic application of anti-CCR5 antibodies in the clinics, substantial bottlenecks need to be overcome for their development and not only for CCR5 but also for G-protein-coupled receptors (GPCRs) in general. GPCRs antigens are difficult to prepare, their extracellular region is very variable, and the number of exposed epitopes is limited (22). GPCRs have been prepared and delivered by different systems (22); however, viral delivery has seldom been investigated.

Taking into account, the promising properties of the BoHV-4 system and following-up to the above studies showing that anti-CCR5 antibodies exert a protective effect against HIV infection, we decided to explore the suitability of BoHV-4 as a vector for the construction of a novel CCR5 immunogen. In this article, we document the capability of an engineered BoHV-4 vector to deliver and express a human CCR5 (hCCR5) expression cassette in rabbits.

## Materials and Methods

### Cells

Bovine embryo kidney [(BS CL-94) BEK, from M. Ferrari, Istituto Zooprofilattico Sperimentale, Brescia, Italy], Dubai Camel cells (ATCC^®^ CRL-2276™, Dubca), BEK-expressing cre recombinase (BEK cre) (3), human embryo kidney 293T [(HEK293T) ATCC: CRL-11268], and alpaca skin stromal cells (23) were cultured in Eagle’s minimal essential medium (EMEM, Lonza) containing 10% fetal bovine serum (FBS), 2 mM l-glutamine (SIGMA), 100 IU/mL penicillin (SIGMA), and 100 µg/mL streptomycin (SIGMA) and 2.5 µg/mL of Amphotericin B (SIGMA) and incubated at 37 C, 5% CO_2_. R5-SupT1-transducted cell line are CCR5-expressing cell lines referred to as SupT1-R5 clone M10 (medium expression of CCR5). The clone was obtained by engineered SupT1 cells and kindly provided by H. Garg (24). The cells (5 × 10^5^) were propagated in complete medium and supplemented with 3 µg/mL of Blasticidin (Calbiochem, Germany).

### Construct Generation

The hCCR5 ORF, was excised from (the commercial plasmid) pcDNA3-hCCR5 (kindly provided by DR. Toon Laeremans, University of Bruxells) cutting with MluI, blunt ended and SmaI restriction enzymes. The ~2,900 bp hCCR5 fragment was then cloned into SmaI cut pINT2 shuttle vector (2) to generate pTK-CMV-hCCR5-TK.

### Transient Transfection

HEK293T cells were seeded into six well plates (3 × 10^5^ cells/well) and incubated at 37°C with 5% CO_2_. When cells were sub-confluent, the culture medium was removed, and the cells were transfected with pTK-CMV-hCCR5-TK using polyethylenimine (PEI) transfection reagent (Polysciences, Inc.). Briefly, 3 µg of DNA was mixed with 7.5 µg PEI (1 mg/mL) (ratio 1:2.5 DNA-PEI) in 200 µL of Dulbecco’s modified essential medium (DMEM) high glucose (Euroclone) without serum. After 15 min at room temperature, 800 µL of medium without serum was added, and the transfection solution was transferred to the cells (monolayer) and left for 6 h at 37°C with 5% CO_2_, in a humidified incubator. The transfection mixture was then replaced with fresh medium EMEM, with 10% FBS, 100 IU/mL of penicillin, 100 µg/mL of streptomycin, and 2.5 µg/mL of Amphotericin B (SIGMA) and incubated for 24 h at 37°C with 5% CO_2_.

### BAC Recombineering and Selection

The PvuI linearized pTK-CMV-hCCR5-TK was used for heat-inducible homologous recombination in SW102 *E. coli* containing the BAC-BoHV-4-A-TK-KanaGalK-TK genome targeted to the TK locus with KanaGalK selector cassette. Recombineering was performed as previously described (25) with some modifications. After recombineering, only those colonies that were kanamycin negative, and chloramphenicol positive were kept and grown overnight in 5 mL of LB containing 12.5 mg/mL of chloramphenicol. Selected SW102 *E. coli* clones carrying pBAC-BoHV-4 recombinants were analyzed by HindIII restriction enzyme digestion. Original detailed protocols for recombineering can also be found at the recombineering website (https://redrecombineering.ncifcrf.gov/background-info/what-is-recombineering.html).

### Southern Blotting

To further confirm our results, a Southern blotting with a probe spanning human CMV promoter sequence, was performed. DNA from 1% agarose gel was capillary transferred to a positively charged nylon membrane (ROCHE), and cross-linked by UV irradiation by standard procedures (3). The membrane was pre-hybridized in 50 mL of hybridization solution (7% SDS, 500 mM sodium phosphate, pH 7.2) for 1 h at 65°C in a rotating hybridization oven (TECHNA INSTRUMENTS).

CMV probe labeled with digoxigenin was generated by PCR with CVM-KpnI-sense (5′-caggggtacctagttattaatagtaatcaat-3′) and CVM-KpnI-antisense (5′-cgcgggtaccgctagcggatctgacggttca 3′) primers, as previously described (4). The PCR amplification reaction was carried out in a final volume of 50 µL, containing 10 mM Tris–hydrochloride pH 8.3, 5% dimethyl sulfoxide, 0.2 mM deoxynucleotide triphosphates, 2.5 mM MgSO_4_, 50 mM KCl, and 0.25 µM of each primer. One hundred nanograms of DNA were amplified over 35 cycles, each cycle consisting of 1 min of denaturation at 94°C, 1 min of primer annealing at 55°C, and 1 min of chain elongation with 1 U of Pfu DNA polymerase (Fermentas) in addition to 1 µL of digoxigenin-11-dUTP, alkali-labile (Roche Life Science) at 72°C.

### Cell Culture Electroporation and Recombinant Virus Reconstitution

Bovine embryo kidney or BEK cre cells were maintained as a monolayer with complete DMEM growth medium with 10% FBS, 2 mM l-glutamine, 100 IU/mL penicillin, and 100 µg/mL streptomycin. When cells were sub-confluent (70–90%), they were split to a fresh culture flask (i.e., every 3–5 days) and were incubated at 37°C in a humidified atmosphere of 95% air, 5% CO_2_. BAC DNA (5 µg) was electroporated in 600 µL DMEM without serum (EQUIBIO APPARATUS, 270 V, 960 mF, 4-mm gap cuvettes) into BEK and BEK cre cells from a confluent 25-cm^2^ flask. Electroporated cells were returned to the flask, after 24 h the medium was replaced with fresh medium, and cells were split 1:2 when they reached confluence at 2 days post-electroporation. Cells were left to grow until the appearance of cytopathic effect (CPE).

### Viruses and Viral Replication

Bovine herpesvirus 4 (BoHV-4)-CMV-hCCR5ΔTK and BoHV-4-A were propagated by infecting confluent monolayers of BEK cells at a multiplicity of infection of 0.5 tissue culture infectious doses 50 (TCID50) per cell and maintained in medium with only 2% FBS for 2 h. The medium was then removed and replaced with fresh EMEM containing 10% FBS. When the CPE affected the majority of the cell monolayer (~72 h post infection), the virus was prepared by freezing and thawing cells three times and pelleting the virions through a 30% sucrose cushion, as described previously (26). Virus pellets were then resuspended in cold EMEM without FBS. TCID50 were determined with BEK cells by limiting dilution.

### Viral Growth Curves

Bovine embryo kidney cells were infected with BoHV-4-A and BoHV-4-CMV-hCCR5ΔTK at a MOI of 0.1 TCID50/cell and incubated at 37°C for 4 h. Infected cells were washed with serum-free EMEM and then overlaid with EMEM containing 10% FBS, 2 mM l-glutamine, 100 IU/mL penicillin, 100 mg/mL streptomycin, and 2.5 mg/mL Amphotericin B. The supernatants of infected cultures were harvested after 24, 48, and 72 h, and the amount of infectious virus was determined by limiting dilution on BEK cells. Viral titer differences between each time point are the averages of triplicate measurements ± SEs of the means (*P* > 0.05 for all time points as measured by Student’s *t*-test).

### Flow Cytometry Assay

To evaluate the presence of hCCR5 protein on the cell surface, a flow cytometry assay was performed on pTK-CMV-hCCR5-TK-transfected HEK293T cells. Cells expressing the protein of interest and mock-transfected cells, which served as negative control, were both assayed with a FITC mouse-antihuman CD195 monoclonal antibody capable of binding to CCR5 Membrane Protein (Clone 2D7/CCR5, Catalog No. 555992, BD Pharmingen™).

The cells plated in a T75 cm^2^ were transfected with 22.5 µg of pTK-CMV-hCCR5-TK DNA, 67.5 µg of PEI (ratio 1:3 DNA-PEI) in 1.5 mL of DMEM high glucose (Euroclone) without serum. After 15 min at room temperature, 6 mL of medium without serum were added, and the transfection solution was transferred to the cells (monolayer) and left for 6 h at 37°C with 5% CO_2_, in a humidified incubator. After 6 h, the transfection mixture was then replaced with fresh medium EMEM, with 10% FBS.

The following day, the transfected cells were firstly briefly washed with sterile phosphate-buffered saline (PBS) to remove any traces of serum and subsequently detached with a PBS–ethylenedinitrilotetraacetic (EDTA) solution (50 µL of EDTA acid 500 mM in a final volume of 50 mL). 2 × 10^5^ resuspended cells, for every sample to test, were centrifuged at 1,200 rpm for 4 min at R.T.

The pelleted cells were incubated at R.T. for 20 min with FITC mouse-antihuman CD195 monoclonal antibody, 1:40 diluted in a final volume of 200 µL of PBS–FBS 2% as suggested by BD Pharmingen. Moreover, 1 MOI of BoHV-4-CMV-hCCR5ΔTK was used to infect BEK, DUBCA, and alpaca cells checking the *in vitro* protein expression on the surface of infected cells by flow cytometry as described before.

Cells surface binding was also performed on Sup-T1-CCR5-M10 cells as previously described (27). Briefly, cells were incubated with 1:10 diluted serum in PBS with 3% FBS from pre-immunized and post-immunized rabbits for 1 h at 4°C, then the cells were washed with PBS and incubated with PE-conjugated goat anti-rabbit antiserum (Sigma-Aldrich) for 30 min at 4°C. Surface expressed CCR5 molecules were detected with anti-CCR5 monoclonal antibody 2D7-PE as the positive control (PE-CF594, mouse-antihuman CD195 monoclonal antibody Becton Dickinson, CA, USA), which has given a mean fluorescence of 40.03. Negative control of binding was done using 5 × 10^5^ cells incubated with PE-conjugated goat anti-rabbit antiserum (Becton Dickinson) for 30 min at 4°C. Ten thousand gated events were acquired using a FACSCalibur flow cytometer (Becton Dickinson). Live cells initially gated by forward and side scatter were analyzed by mean fluorescence intensity (MFI). MFI of each sample was calculated by subtracting MFI of negative control. The results are given as mean of MFI of two independent experiments.

### Animal Handling and Vaccination

Rabbits were cared for and used in accordance with Italian laws for animal experimentation. Rabbits were maintained at 24°C with a controlled light cycle (12 h of light, starting at 6:00 a.m.) and with food and water *ad libitum*. Blood samples were obtained, and viral injections were performed *via* the auricular vein at scheduled intervals. In agreement with the current legislation on animal experimentation, which suggests to minimize the number of animals employed, two adult rabbits, after collection of the preimmune serum, were inoculated intravenously with 1 mL of 10^5^ TCID50 of BoHV-4-CMV-hCCR5ΔTK. A second inoculation with an identical dose of BoHV-4-CMV-hCCR5ΔTK was done 2 weeks apart from the first inoculation. Blood samples were collected at 2 weeks, just before the second inoculum, and 5 weeks from the first inoculum. The experiments comply with the Principles of Animal Care (NIH Publication no. 85-23. Revised 1985) of the National Institutes of Health and with the current law of the European Union and Italy. The present project was approved by the Ethical Committee of the University of Parma (OPBA: prot. n. 49/13 del 08/07/2013).

### Immunofluorescence Antibody Test (IFAT) Assay

To evaluate the presence of anti-CCR5 antibody, an immunofluorimetric assay was performed on pTK-CMV-hCCR5-TK-transfected and mock-transfected HEK293T cells and treated with the serum of BoHV-4-CMV-hCCR5ΔTK-vaccinated rabbits.

The cells plated in a T75 cm^2^ were transfected with 22.5 µg of pTK-CMV-hCCR5-TK DNA, 67.5 µg of PEI (ratio 1:3 DNA-PEI) in 1.5 mL of DMEM high glucose (Euroclone) without serum. After 15 min at room temperature, 6 mL of medium without serum was added, and the transfection solution was transferred to the cells (monolayer) and left for 6 h at 37°C with 5% CO_2_, in a humidified incubator. After 6 h, the transfection mixture was then replaced with fresh medium EMEM, with 10% FBS.

24 h after the transfection, the cells were firstly briefly washed with sterile PBS to remove any traces of serum and subsequently detached with sterile PBS. After three PBS washes, the cells were resuspended in PBS plus rabbit serum diluted to 1:20; preimmune and 5 weeks post vaccination serum were tested. After 1 h at R.T. incubation, cells were washed in PBS three times, and the cells were resuspended in PBS plus 1:40 FITC-conjugated goat anti-rabbit IgG (F0382, SIGMA) secondary antibody. After 30 min of incubation in the dark at 4°C, the samples were PBS washed three times, resuspended in PBS and visualized on a plastic chamber slide with the fluorescence microscope Axiovert S100 (Zeiss). Images were achieved using a digital CCD camera, and images were processed using the AxioVision 40-V4.6.3.0 (Carl Zeiss, Imaging Solution) software program.

### Synthesis of Peptides

Peptides were synthesized by the solid-phase Fmoc method (28) using an Applied Biosystems model 433A peptide synthesizer. After peptide assembly, resin-bound peptides were deprotected as previously described (29) and purified to greater than 95% purity by semipreparative reverse-phase high-performance liquid chromatography. To obtain conformationally restricted etherocyclic peptides, an extra cysteine was added at peptides covering ECL1 extracellular regions as previously described (19–21). All extracellular regions of CCR5 have been synthesized as shown in Table 1 and in particular amino-terminal region from aa 14 to 34 as it has been demonstrated to be immunogenic whereas peptide from 1 to 13 did not induce antibodies (20, 21); ECL1 as constrained peptide, as its linear form did not elicit antibodies (19–21); ECL2 and ECL3 regions in their linear sequence.

**Table 1:** Binding of CCR5-specific rabbit antisera (#1 and #2) to extracellular region peptides of CCR5 and to CCR5 expressed by the cell line Sup-T1-l.

Assays	Peptides covering CCR5 extramembrane regions	Sequence	Rabbit antiserum #1	Rabbit antiserum #2
			Pre–post immunization	Pre–post immunization
ELISA^a^	N terminus (aa 14–34)	YYTSEPCQKINVKQIAARLLP	<20–540	<20–180
	ECL1(aa 89–102)	YAAAQWDFGNTMCQ	<20–<20	<20–<20
	ECL2 (aa 178–197)	CSSHFPYSQYQFWKN FQTLK	<20–<20	<20–<20
	ECL3 (aa 260–274)	FQFFGLNNCSSSNR	<20–<20	<20–<20
	Biotinylated constrained ECL1 (aa 89–103)^b^	kgcYAAAQWDFGNTMCQgk	<20–<20	<20–<20
Flow cytometry^c^			2.4–9.14	2.7–13.17

*^a^ELISA results are expressed as titers (serum dilution 1/*n*)*.

*^b^Constrained peptide has been obtained introducing an extra cystein*.

*^c^Flow cytometry results are expressed as mean fluorescence intensity*.

### CCR5-Specific ELISA

To quantify the rabbit serum antibodies against CCR5, microwell plates were coated with synthetic peptides covering the extra membrane regions of CCR5 (amino-terminal, ECL1, ECL2, and ECL3 as shown in Table 1), at 0.1 µg/well in 50 mM carbonate buffer, pH 9.5, for 1 h at 37°C. The plates were saturated for 1 h with 1% milk (Sigma-Aldrich, MO, USA) in PBS. Serial dilutions (from 1:20 up to 1:4,860 by threefold dilutions) of pre-immunized and post-immunized specific antisera were added to 1% milk and 0.1% Tween 20 (Sigma-Aldrich) in PBS. Then, peroxidase-conjugated goat anti-rabbit total IgG (Sigma-Aldrich) was added and incubated for 30 min at 37°C. The enzymatic reaction was developed with the TMB Microwell Peroxidase Substrate System (KPL, MD, USA) and read in a plate reader (Biotek, VT, USA) at 492 nm. The reciprocal endpoint titers were determined as the last sample dilution that produced a threefold greater absorbance than the ones of the sample diluent and expressed at serum dilution 1/*n*. Two independent experiments have been performed, and results are expressed as mean value of titers. To quantify antibodies directed to conformational epitope of ECL1, avidin (Sigma-Aldrich) was coated at 1.5 µg/well in 50 mM carbonate buffer, pH 9.5 and then biotynilated-ECL1 constrained peptide was used. The rest of the assay was similar as for the ELISA performed with the other CCR5 peptides but using 10% bovine serum albumin (Sigma-Aldrich), 30% fetal calf serum from Lonza, Belgium, and 0.1% Tween 20 in PBS as either blocking or diluent buffer (19).

## Results and Discussion

**Figure 1:**
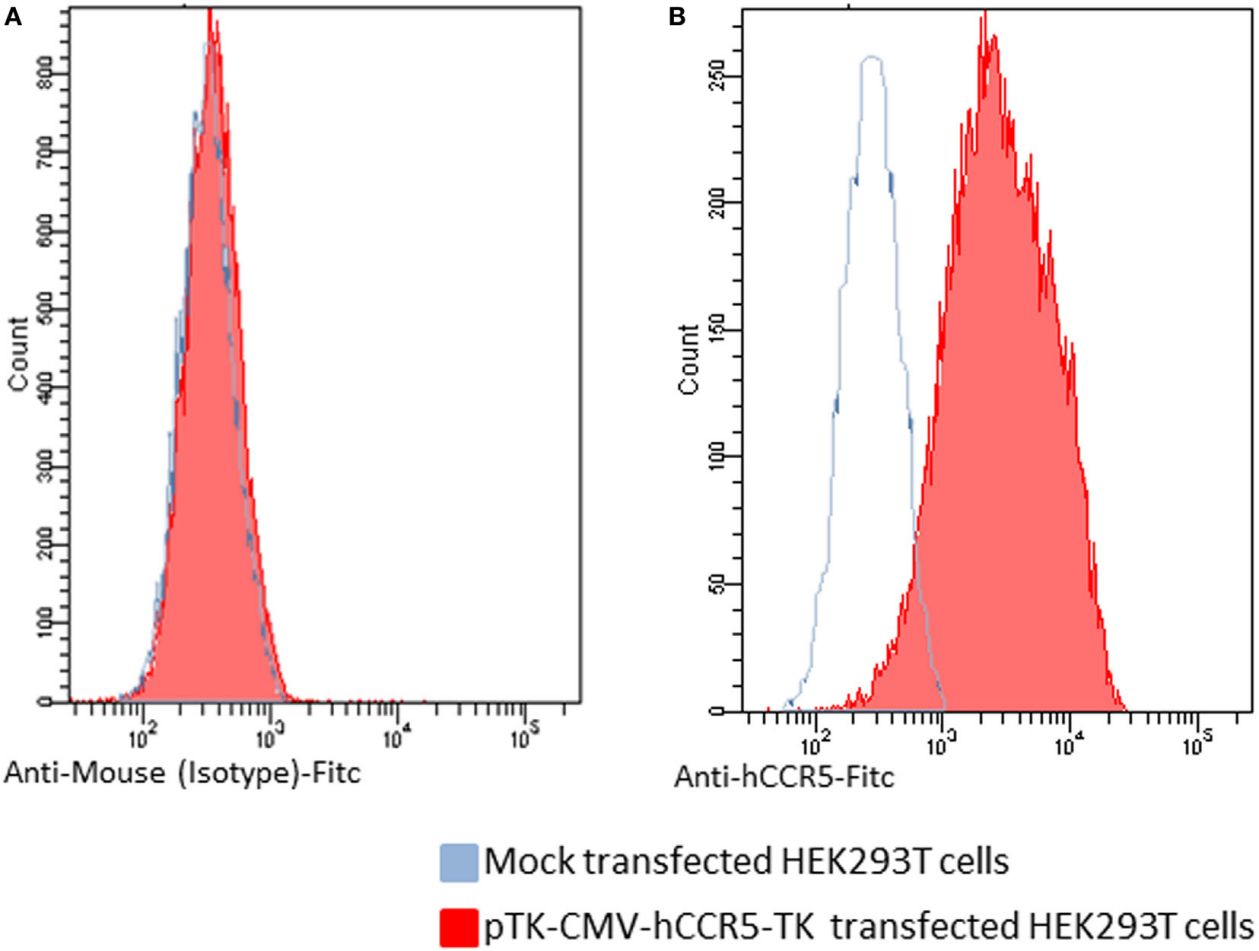
Flow cytometric analysis of human CCR5 (hCCR5) on the cell surface of pTK-CMV-hCCR5-TK-transfected HEK293T cells. The blue line corresponds to mock-transfected cells, and the red line corresponds to the pTK-CMV-hCCR5-TK-transfected cells. **(A)** To analyze pTK-CMV-hCCR5-TK- and mock-transfected cells, a FITC-conjugated anti-isotype antibody, which served as negative control antibody, was assayed. **(B)** To analyze pTK-CMV-hCCR5-TK and mock-transfected cells, a FITC-conjugated anti-hCCR5 antibody was used. The consistent shift of the red lane indicates the cell surface staining of pTK-CMV-hCCR5-TK transfected cells with respect to the mock-transfected control.

The first step of this study was to generate a recombinant BoHV-4 derivative capable of delivering a hCCR5 expression cassette. Starting from a pCMV-hCCR5 plasmid vector containing the hCCR5 ORF under the control of the CMV promoter and the bovine growth hormone polyadenylation signal (BGHpA), the entire expression cassette (CMV-hCCR5-BGHpA) was excised by restriction digestion and sub-cloned into the pINT2 shuttle vector. pINT2 contains two BoHV-4 TK flanking sequences (1) that were used to generate the targeting vector pTK-CMV-hCCR5-TK. In fact, the BoHV-4 TK genomic locus can harbor foreign DNA sequences of varying length (up to 20 kbp) without any impairment of *in vitro* viral replication nor expression of the corresponding heterologous proteins. hCCR5 expression was functionally proved by pTK-CMV-hCCR5-TK HEK transient transfection into 293 T cells and by immunoblotting and flow cytometry detection using an anti-hCCR5 monoclonal antibody. As shown in Figures 1A,B, hCCR5 was well expressed on the surface of transfected HEK293T cells.

**Figure 2:**
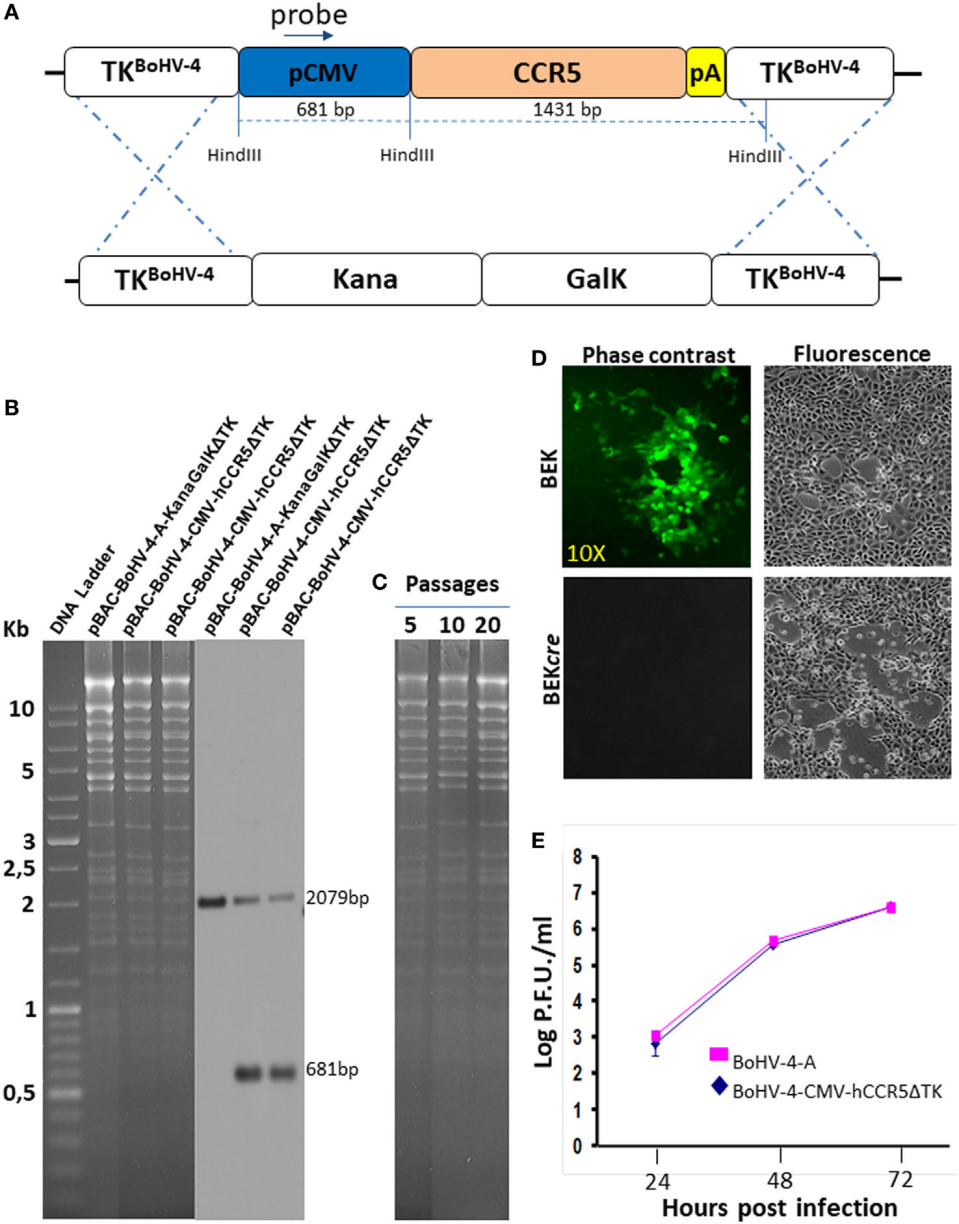
Recombinant bovine herpesvirus 4 (BoHV-4) expressing human CCR5 (hCCR5). **(A)** Heat-inducible homologous recombination in SW102 containing pBAC-BoHV-4-A-TK-KanaGalK-TK, where the Kana/GalK cassette was substituted with the CMV-hCCR5-BGHpA expression cassette was depicted (not to scale). **(B)** Putative positive colonies were controlled by HindIII restriction enzyme analysis, agar gel electrophoresis, and Southern blotting performed with a probe for the CMV promoter sequence, where the 681 bp band is present only for the targeted clones but not for the untargeted control. The 2,079 pb band corresponds to the CMV promoter driving the GFP expression and contained within the bacterial artificial chromosome (BAC) cassette in both targeted and untargeted clones. **(C)** Stability of the pBAC-BoHV-4-CMV-hCCR5ΔTK plasmid in *E. coli* SW102 cells was tested until the 20th passage. **(D)** Representative fluorescent microscopic images of plaques formed by viable reconstituted recombinant BoHV-4-CMV-hCCR5ΔTK after DNA electroporation into bovine embryo kidney (BEK) cells or in BEK cells expressing cre recombinase. Magnification, 10×. **(E)** Replication kinetics of BoHV-4-CMV-hCCR5ΔTK growth on cre-expressing cells, compared with those of the parental BoHV-4-A isolate. The data presented are the means ± SEs of triplicate measurements (*P* > 0.05 for all time points as measured by Student’s *t*-test).

Restriction enzyme linearized pTK-CMV-hCCR5-TK was then recombined into pBAC-BoHV-4-A-KanaGalKΔTK contained in SW102 *E. coli* cells by heat-inducible homologous recombination, to generate pBAC-BoHV-4-CMV-hCCR5ΔTK (Figure 2A). HindIII digestion and Southern blotting were performed to analyze selected clones (Figure 2B). Because altered bacterial phenotypes due to aberrant recombinase transcription can be generated through heat-inducible recombination and repeated passages in bacteria, SW102 *E. coli* cells carrying pBAC-BoHV-4-CMV-hCCR5ΔTK were serially cultured for 20 passages and the corresponding BAC DNAs were repeatedly checked by HindIII digestion analysis. Throughout the various passages, no restriction pattern differences were detected (Figure 2C), thus confirming clone stability.

To reconstitute infectious viral particles, pBAC-BoHV-4-CMV-hCCR5ΔTK was electroporated into BEK and BEK*cre* cells for the excision of BAC cassette. Both cellular lines allowed viable virus reconstitution (Figure 2D) and as attended the loss of the green fluorescence was observed in the viral progeny indicating the excision of the CMV-GFP expression cassette within the floxed BAC plasmid backbone. Since reconstitution of viable BoHV-4-CMV-hCCR5ΔTK required a longer time than reconstitution of the parental BoHV-4-ΔTK (3 and 4 days, respectively), it was of interest to know if hCCR5, encoded by the expression cassette integrated into the viral genome, might have a detrimental effect on viral replication. As shown in Figure 2E, no significant difference was observed between the growth (replication) performance of the recombinant BoHV-4-CMV-hCCR5ΔTK virus and the empty BoHV-4-ΔTK controls, thus indicating the replication competence of the BoHV-4-A BAC derivative containing the CMV-hCCR5-BGHpA expression cassette.

**Figure 3:**
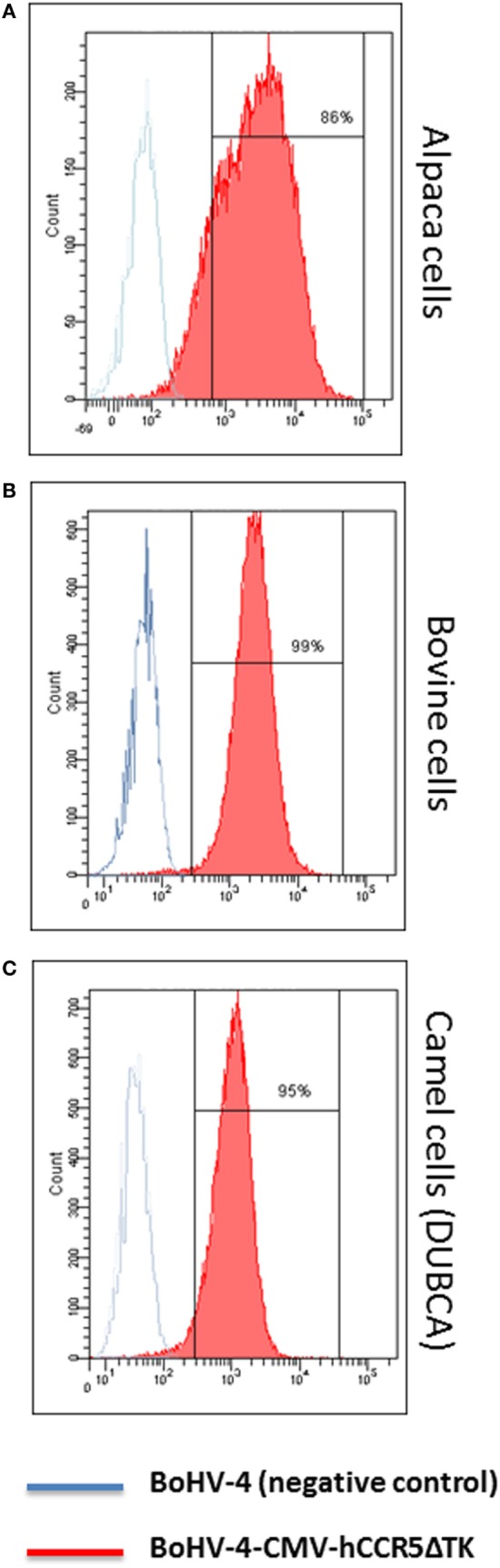
Flow cytometric analysis of bovine herpesvirus 4 (BoHV)-4-CMV-hCCR5ΔTK infected cells. Alpaca **(A)**, bovine **(B)**, and camel **(C)** cell lines expressing human CCR5 (hCCR5) on their surface were analyzed. The blue line corresponds to BoHV-4 infected, used as a negative control, whereas the red line corresponds to BoHV-4-CMV-hCCR5ΔTK infected cells. The consistent shift of the red lane indicates the cell surface staining of BoHV-4-CMV-hCCR5ΔTK-infected cells respect to the mock-transfected control. Efficiency of infection/transduction is indicated, 86, 99, and 95% for alpaca, bovine, and camel, respectively. Anti-isotype antibody control gave a complete overlapping of the two peaks for the three cell lines tested.

More importantly, cells from different artiodactyl infected with BoHV-4-CMV-hCCR5ΔTK and assayed by flow cytometry, robustly expressed membrane-associated hCCR5 (Figure 3).

**Figure 4:**
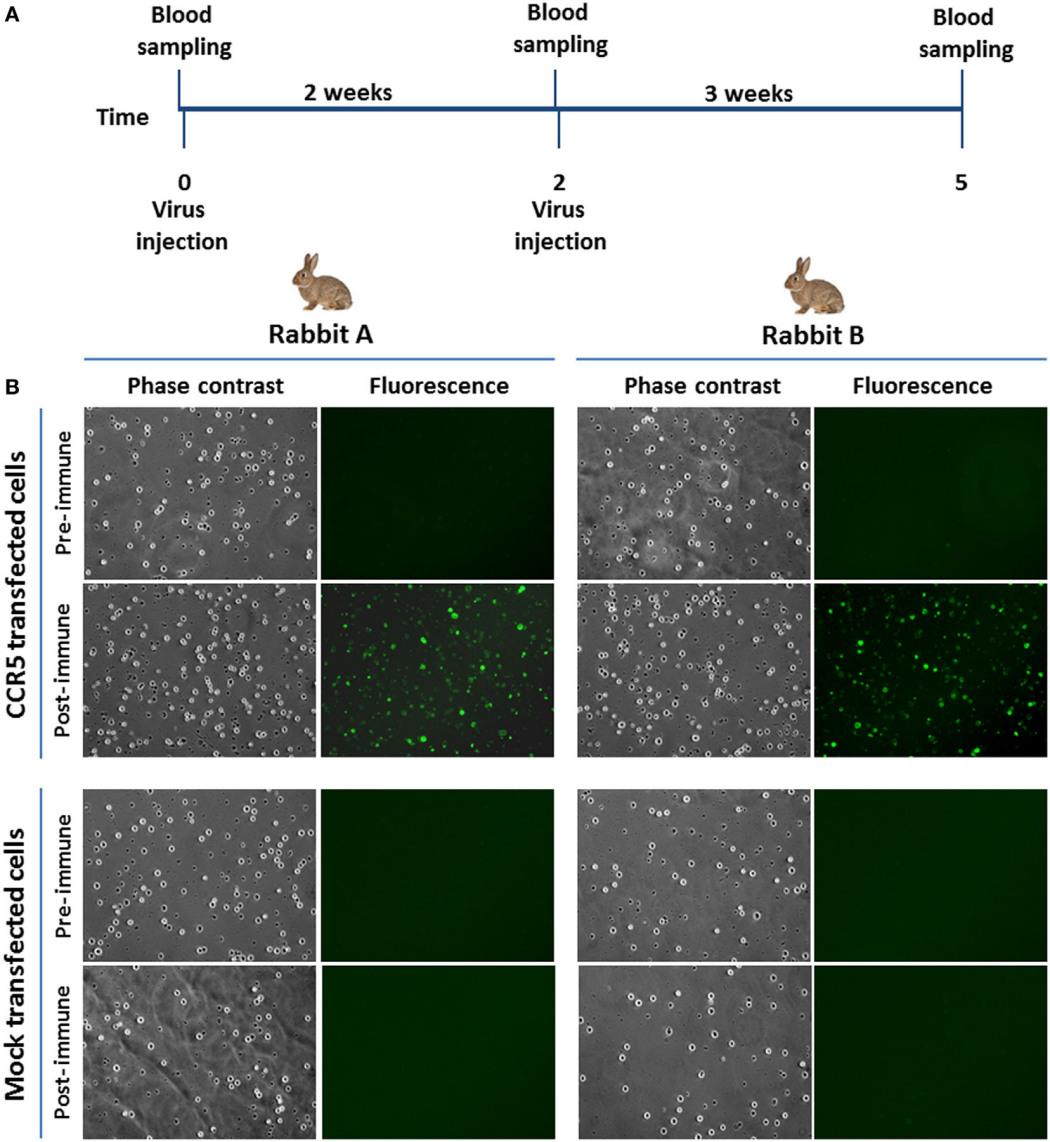
Rabbit immunization. **(A)** Diagram showing rabbit immunization scheme and blood sample collection. **(B)** Representative microscopic immunofluorescence antibody test images (magnification, 10×) of HEK293T cells transfected with pTK-CMV-human CCR5 (hCCR5)-TK or mock transfected and challenged with sera from BoHV-4-CMV-hCCR5ΔTK-immunized rabbits. The presence of anti-hCCR5 antibodies in the rabbit serum samples (5 weeks post immunization) is detectable by green cells when observed with FITC filter. Negative controls were established with preimmune sera.

Next, immunogenicity of BoHV-4-CMV-hCCR5ΔTK was tested in rabbits. In accordance with European laws, which emphasize minimizing the number of animals utilized for pre-clinical experimentation, BoHV-4-CMV-hCCR5ΔTK was inoculated into only two rabbits. 1 mL of 10^5^ TCID_50_/mL of BoHV-4-CMV-hCCR5ΔTK was intravenously inoculated into adult rabbits, after preimmune serum collection. 2 weeks after the first immunization the inoculation was identically repeated. Collection of blood samples was performed 3 weeks after the last inoculation, at which time none of the animals developed fever or other adverse effects attributable to immunization (Figure 4A) and this was in agreement with previously published data (4). As demonstrated by an IFAT conducted on pTK-CMV-hCCR5-TK-transfected HEK293T cells (Figure 4B), both immunized animals mounted an anti-hCCR5 antibody response.

Furthermore, as revealed by hCCR5 peptide mapping in ELISA, the humoral immune response elicited by BoHV-4-CMV-hCCR5ΔTK was directed against the hCCR5 amino-terminal region comprised between amino acids (aa) 14–34, which represents the most immunogenic region in this experimental setting (Table 1).

Alterations of self-antigens can be induced by viral infection rising auto-immunogenic proteins and their corresponding autoantibodies. Conformational changes in host receptors and self-proteins reshaping of non-self-antigenic epitopes can occur in response to host factors or other latent or concomitant viral infections, causing perturbations in host cells (30).

Previous immunization experiments in mice have demonstrated the feasibility of breaking self-tolerance and inducing significant levels of anti-CCR5 antibodies with the use of appropriate, non-live recombinant antigens (20, 31). In this context, live virus-based antigen formulation systems deserve special attention because of their relative ease of production and ability to deliver the antigen expression cassette directly into host cells, thus allowing high-level expression of the antigen as well as a widespread distribution of transduced cells, ultimately leading to a superior immunogenicity *via* a cross-priming mechanism. It should be noted, however, that the immune system has evolved multiple mechanisms to detect and eliminate invading viruses and that different viral vector systems may be differently suited for diverse and specific immunization purposes. Considering this, the viral vector can be used as an adjuvant and a delivery system. To stimulate an optimized immune response, an efficient viral vector should remain in the host organism long enough to express the antigen as an immune target. Preexistence of anti-vector immunity in the host organism is one of the major obstacle for the development of a vaccine vector. BoHV-4 is able to bypass this barrier because it does not naturally stimulate serum neutralizing antibodies production. Compared to DNA vaccines, virus vaccines are more stably storable and useful for multiple purposes. Moreover, DNA-based vaccines present lower relative efficacy, requiring high doses of multiple boosts (up to 500 µg of plasmid DNA per injection) in order to obtain similar responses to those of attenuated virus vaccination.

Given the unpredictable performance of different viruses as antigen carriers, experimental testing of individual viruses is required to identify the best suited vaccine vector agent. The data presented in this pilot-study, which clearly point to the potential of BoHV-4 as a gene delivery vector capable of conferring immunogenicity to poorly antigenic (and self) proteins such as hCCR5, represent a first step in this direction. This innovative approach, together with other immune-modulating strategies, could lead to new treatment perspectives both for HIV/AIDS and other disorders characterized by detrimental pro-inflammatory responses.

## Availability of Data and Material

Available under request.

## Ethics Statement

Animal experiments were conducted in compliance with national (Decreto Legislativo numero 26, 4 Marzo 2014) and international laws and policies (Guide for the Care and Use of Laboratory Animals). The present project was approved by the Ethical Committee of the University of Parma (OPBA: prot. n. 49/13 del 08/07/2013).

## Author Contributions

GD conceived the experiments and wrote the paper. AV, VF, GT, FM, VM, CP, and GD performed the experiments. GD, LL, SC, and SO analyzed the data.

## Conflict of Interest Statement

The authors declare that the research was conducted in the absence of any commercial or financial relationships that could be construed as a potential conflict of interest.

## Abbreviations

BoHV-4, bovine herpesvirus 4; CCR5, C–C chemokine receptor type 5; BAC, bacterial artificial chromosome; HSV-1-TK, herpes simplex virus-1 thymidine kinase; HIV, human immunodeficiency virus; BEK, bovine embryo kidney; Dubca, Dubai camel cells; HEK293T, human embryo kidney 293T; ASSC, alpaca skin stromal cells; FBS, fetal bovine serum; DMSO, dimethyl sulfoxide; MOI, multiplicity of infection; TCID50, tissue culture infectious doses 50; EDTA, ethylenedinitrilotetraacetic; MFI, mean fluorescence intensity.

## References

[1] Donofrio G Cavirani S Simone T van Santen VL. Potential of bovine herpesvirus 4 as a gene delivery vector. J Virol Methods (2002) 101(1–2):49–61. 10.1016/S0166-0934(01)00419-0 11849683

[2] Donofrio G Sartori C Ravanetti L Cavirani S Gillet L Vanderplasschen A Establishment of a bovine herpesvirus 4 based vector expressing a secreted form of the bovine viral diarrhoea virus structural glycoprotein E2 for immunization purposes. BMC Biotechnol (2007) 7:68. 10.1186/1472-6750-7-68 17945009 PMC2048506

[3] Donofrio G Sartori C Franceschi V Capocefalo A Cavirani S Taddei S Double immunization strategy with a BoHV-4-vectorialized secreted chimeric peptide BVDV-E2/BoHV-1-gD. Vaccine (2008) 26(48):6031–42. 10.1016/j.vaccine.2008.09.023 18812200

[4] Donofrio G Franceschi V Capocefalo A Taddei S Sartori C Bonomini S Cellular targeting of engineered heterologous antigens is a determinant factor for bovine herpesvirus 4-based vaccine vector development. Clin Vaccine Immunol (2009) 16(11):1675–86. 10.1128/CVI.00224-09 19793901 PMC2772392

[5] Franceschi V Capocefalo A Calvo-Pinilla E Redaelli M Mucignat-Caretta C Mertens P Immunization of knock-out alpha/beta interferon receptor mice against lethal bluetongue infection with a BoHV-4-based vector expressing BTV-8 VP2 antigen. Vaccine (2011) 29(16):3074–82. 10.1016/j.vaccine.2011.01.075 21320537

[6] Redaelli M Franceschi V Capocefalo A D’Avella D Denaro L Cavirani S Herpes simplex virus type 1 thymidine kinase-armed bovine herpesvirus type 4-based vector displays enhanced oncolytic properties in immunocompetent orthotopic syngenic mouse and rat glioma models. Neuro Oncol (2012) 14(3):288–301. 10.1093/neuonc/nor219 22228853 PMC3280804

[7] Donofrio G Franceschi V Lovero A Capocefalo A Camero M Losurdo M Clinical protection of goats against CpHV-1 induced genital disease with a BoHV-4-based vector expressing CpHV-1 gD. PLoS One (2013) 8(1):e52758. 10.1371/journal.pone.0052758 23300989 PMC3536792

[8] Franceschi V Parker S Jacca S Crump RW Doronin K Hembrador E BoHV-4-based vector single heterologous antigen delivery protects STAT1(-/-) mice from Monkeypox virus lethal challenge. PLoS Negl Trop Dis (2015) 9(6):e0003850. 10.1371/journal.pntd.0003850 26086739 PMC4473039

[9] Jacca S Rolih V Quaglino E Franceschi V Tebaldi G Bolli E Bovine herpesvirus 4-based vector delivering a hybrid rat/human HER-2 oncoantigen efficiently protects mice from autochthonous Her-2+ mammary cancer. Oncoimmunology (2016) 5(3):e1082705 10.1080/2162402X.2015.1082705 27141335 PMC4839386

[10] Donofrio G Martignani E Poli E Lange C Martini FM Cavirani S Bovine herpesvirus 4 based vector interaction with liver cells in vitro and in vivo. J Virol Methods (2006) 136(1–2):126–36. 10.1016/j.jviromet.2006.04.008 16712963

[11] Donofrio G Taddei S Franceschi V Capocefalo A Cavirani S Martinelli N Swine adipose stromal cells loaded with recombinant bovine herpesvirus 4 virions expressing a foreign antigen induce potent humoral immune responses in pigs. Vaccine (2011) 29(5):867–72. 10.1016/j.vaccine.2010.11.048 21115049

[12] Lederman MM Penn-Nicholson A Cho M Mosier D. Biology of CCR5 and its role in HIV infection and treatment. JAMA (2006) 296(7):815–26. 10.1001/jama.296.7.815 16905787

[13] Lopalco L Barassi C Pastori C Longhi R Burastero SE Tambussi G CCR5-reactive antibodies in seronegative partners of HIV-seropositive individuals down-modulate surface CCR5 in vivo and neutralize the infectivity of R5 strains of HIV-1 In vitro. J Immunol (2000) 164(6):3426–33. 10.4049/jimmunol.164.6.3426 10706739

[14] Barassi C Lazzarin A Lopalco L CCR5-specific mucosal IgA in saliva and genital fluids of HIV-exposed seronegative subjects. Blood (2004) 104(7):2205–6. 10.1182/blood-2004-06-2134 15377578

[15] Pastori C Weiser B Barassi C Uberti-Foppa C Ghezzi S Longhi R Long-lasting CCR5 internalization by antibodies in a subset of long-term nonprogressors: a possible protective effect against disease progression. Blood (2006) 107(12):4825–33. 10.1182/blood-2005-06-2463 16522810 PMC1895813

[16] Bomsel M Pastori C Tudor D Alberti C Garcia S Ferrari D Natural mucosal antibodies reactive with first extracellular loop of CCR5 inhibit HIV-1 transport across human epithelial cells. AIDS (2007) 21(1):13–22. 10.1097/QAD.0b013e328011049b 17148963

[17] Chackerian B Lowy DR Schiller JT. Induction of autoantibodies to mouse CCR5 with recombinant papillomavirus particles. Proc Natl Acad Sci U S A (1999) 96(5):2373–8. 10.1073/pnas.96.5.2373 10051649 PMC26791

[18] Olson WC Rabut GE Nagashima KA Tran DN Anselma DJ Monard SP Differential inhibition of human immunodeficiency virus type 1 fusion, gp120 binding, and CC-chemokine activity by monoclonal antibodies to CCR5. J Virol (1999) 73(5):4145–55. 10196311 10.1128/jvi.73.5.4145-4155.1999 PMC104194

[19] Barassi C Soprana E Pastori C Longhi R Buratti E Lillo F Induction of murine mucosal CCR5-reactive antibodies as an anti-human immunodeficiency virus strategy. J Virol (2005) 79(11):6848–58. 10.1128/JVI.79.11.6848-6858.2005 15890924 PMC1112124

[20] Pastori C Clivio A Diomede L Consonni R De Mori GM Longhi R Two amino acid substitutions within the first external loop of CCR5 induce human immunodeficiency virus-blocking antibodies in mice and chickens. J Virol (2008) 82(8):4125–34. 10.1128/JVI.02232-07 18256149 PMC2293020

[21] Pastori C Diomede L Venuti A Fisher G Jarvik J Bomsel M Induction of HIV-blocking anti-CCR5 IgA in Peyers’s patches without histopathological alterations. J Virol (2014) 88(7):3623–35. 10.1128/JVI.03663-13 24403594 PMC3993546

[22] Jo M Jung ST. Engineering therapeutic antibodies targeting G-protein-coupled receptors. Exp Mol Med (2016) 48:e207. 10.1038/emm.2015.105 26846450 PMC4892866

[23] Franceschi V Jacca S Sassu EL Stellari FF van Santen VL Donofrio G. Generation and characterization of the first immortalized alpaca cell line suitable for diagnostic and immunization studies. PLoS One (2014) 9(8):e105643. 10.1371/journal.pone.0105643 25140515 PMC4139384

[24] Joshi A Garg H Ablan SD Freed EO. Evidence of a role for soluble N-ethylmaleimide-sensitive factor attachment protein receptor (SNARE) machinery in HIV-1 assembly and release. J Biol Chem (2011) 286(34):29861–71. 10.1074/jbc.M111.241521 21680744 PMC3191027

[25] Warming S Costantino N Court DL Jenkins NA Copeland NG. Simple and highly efficient BAC recombineering using galK selection. Nucleic Acids Res (2005) 33(4):e36. 10.1093/nar/gni035 15731329 PMC549575

[26] Donofrio G Cavaggioni A Bondi M Cavirani S Flammini CF Mucignat-Caretta C Outcome of bovine herpesvirus 4 infection following direct viral injection in the lateral ventricle of the mouse brain. Microbes Infect (2006) 8(3):898–904. 10.1016/j.micinf.2005.10.016 16503181

[27] Mettler Izquierdo S Varela S Park M Collarini EJ Lu D Pramanick S High-efficiency antibody discovery achieved with multiplexed microscopy. Microscopy (Oxf) (2016) 65(4):341–52. 10.1093/jmicro/dfw014 27107009 PMC5895110

[28] Fields GB Noble RL Solid phase peptide synthesis utilizing 9-fluorenylmethoxycarbonyl amino acids. Int J Pept Protein Res (1990) 35(3):161–214. 10.1111/j.1399-3011.1990.tb00939.x 2191922

[29] King DS Fields CG Fields GB. A cleavage method which minimizes side reactions following Fmoc solid phase peptide synthesis. Int J Pept Protein Res (1990) 36(3):255–66. 10.1111/j.1399-3011.1990.tb00976.x 2279849

[30] Lopalco L. Natural anti-CCR5 antibodies in HIV-infection and -exposure. J Transl Med (2011) 9(Suppl 1):S4. 10.1186/1479-5876-9-S1-S4 21284903 PMC3105504

[31] Lopalco L. CCR5: from natural resistance to a new anti-HIV strategy. Viruses (2010) 2(2):574–600. 10.3390/v2020574 21994649 PMC3185609

